# A modified, hypoallergenic variant of the *Ricinus communis* Ric c1 protein retains biological activity

**DOI:** 10.1042/BSR20171245

**Published:** 2018-03-16

**Authors:** Thaís Pacheco-Soares, André de Oliveira Carvalho, Jucélia da Silva Araújo, Giliane da Silva de Souza, Olga L.T. Machado

**Affiliations:** 1 Laboratório de Química e Função de Proteínas e Peptídeos (LQFPP), Universidade Estadual do Norte Fluminense Darcy Ribeiro, Campos dos Goytacazes, RJ 2000, Brazil; 2 Laboratório de Fisiologia e Bioquímica de Mico-organismos (LFBM), Universidade Estadual do Norte Fluminense Darcy Ribeiro, Campos dos Goytacazes, RJ 2000, Brazil; Laboratório Biologia do Reconhecer (LBR), 3 Universidade Estadual do Norte Fluminense Darcy Ribeiro, Campos dos Goytacazes, RJ 2000, Brazil

**Keywords:** allergies, hypoallergern, immunoterapy, mutation, Ricinus communis

## Abstract

Ric c1, an allergenic protein from castor oil plants (*Ricinus communis*), is an insect α-amylase inhibitor that has become an occupational allergen. Ric c1 can cross-react with allergens from wheat, soybean, peanut, shrimp, fish, gluten, house dust, tobacco and air fungus, thereby amplifying the concern and risks caused by castor oil plants (COP) allergens. Two continuous IgE-binding epitopes were identified in Ric c1, both containing glutamic acid residues involved in IgE-binding and allergic challenges. We produced recombinant Ric c1 (rRic c1) in *Escherichia coli*, using primers from foliar castor oil plant DNA, and a mutant (Glu-Leu) recombinant protein (mrRic c1) in the same system using synthetic genes. rRic c1 preserved both allergenic and α-amylase inhibitory properties, and mrRic c1 drastically reduced allergenic properties. These results can help to establish meaningful relationships between structure, defence and allergenicity, important steps for producing engineered plants and developing new approaches for immunotherapy.

## Introduction

An allergy is commonly defined as a type I immediate hypersensitivity reaction, where the symptoms appear quickly and are caused by exposure to exogenous macromolecules, known as antigens or allergens, usually proteins or peptides [1,2]. Allergic responses are usually mediated by immunoglobulin E (IgE). The cross-linking of IgE associated with FcϵRI in mast-cell and basophilic membranes by allergens is a key event in acute allergic inflammation [3]. It induces the immediate release of inflammatory mediators (histamine and leukotrienes), which are responsible for the most common manifestations of type I allergies. The global prevalence of allergies has been continuously rising in recent decades, and currently more than 25% of the population is affected [3]. Symptoms of an allergy can be diverse and observed in the skin, respiratory system, gastrointestinal tract, and the systemic manifestations including anaphylactic shock. Allergen-specific immunotherapy (ASIT) uses recombinant allergens and is one of the few antigen-specific treatments for inflammatory diseases [4]. Wild-type allergens are native allergens and are generally similar in structure and immunological properties. Hypoallergenic recombinant allergens can be produced by mutating B- and T-cell epitopes or by altering IgE epitopes, reducing their allergenicity [3].

The Ric c1 and Ric c3 allergenic proteins in castor oil seeds are 2S albumin isoforms that function as storage and defensive proteins. 2S albumin from *Ricinus* contains many glutamine residues, has a molecular mass of 11–15 kDa [5] and comprises two polypeptide chains linked by two disulphide bridges. Ric c1 and Ric c3 are synthesized from a single precursor with 237 amino acid residues [5]. 2S albumins in seeds are found in protein-storage vacuoles and are mobilized during germination, acting as nitrogen and sulphur donors [6]. The defensive functions of 2S albumins include the inhibition of serine proteases [7] and the α-amylases of the larval insects *Tenebrio molitor, Callosobruchus maculatus* and *Zabrotes subfasciatus* [8]; despite these important functions in seeds, Ric c1 and Ric c3 are allergenic proteins [8,6] that represent risks to the health of farm workers and the distributors of castor seeds who are constantly exposed to the allergens. Castor allergens are included amongst occupational allergens [9].

Cross-reactions between Ric c1 and allergens have been reported for corn, wheat, soybean, peanut, shrimp, fish, house dust, tobacco and airborne fungi, amplifying the concern and risks caused by *Ricinus* allergens [10]. The development of more effective, safe, convenient, broadly applicable and easily manufactured vaccines for ASITs has been limited for many years by the poor quality of extracts of allergens. Expression of the genes encoding the proteins can be a better alternative to obtaining isolated proteins, due to the presence of isoforms with similar physical properties [11]. A single precursor is often post-translationally processed by endopetidases and carboxypeptidases, losing some peptide fragments to generate isoforms and producing more than one 2S albumin isoform, as observed in castor oil seeds [12]. Detailed information of the characteristics of food and airborne allergens and their structure, biological activity and stability may be helpful for improving the diagnosis of allergies by avoiding unnecessary dietary exclusions and for assessing the risk of cross-reactive allergies to other food sources or from air exposure.

We first report the production of recombinant Ric c1 (rRic c1) as a single polypeptide chain (including a linker peptide) in a heterologous prokaryotic *Escherichia coli* expression system and compare its biological activity to that of native Ric c1 (nRic c1). We then demonstrate that important biological activities such as α-amylase inhibition and allergenicity can be preserved with this expression model, facilitating studies not only with castor oil plant allergens, but probably also with several other allergens in other food sources and for aeroallergens.

Felix et al. [13] and Deus de Oliveira et al. [10] identified six epitopes in castor oil seeds (COS) allergens responsible for the allergic trigger, two in Ric c1 and four in Ric c3 [10,13]. Two residues of glutamic acid in each IgE-binding epitope are important for binding the allergen with IgE previously fixed on mast-cell and/or basophilic membranes, inducing cellular degranulation and releasing mediators that lead to the allergic symptoms [10,13]. Point mutations in the DNA coding for these amino acids involved in the interaction between IgE and the allergen were introduced, producing a hypoallergenic protein that retained α-amylase inhibition, as predicted by *in silico* studies [8]. We used this information to subsequently obtain a mutant recombinant hypoallergenic protein (mrRic c1).

## Material and methodology

### Biological material and animals

*R. communis* L. (castor oil seeds), cultivar IAC-226, was obtained from the Instituto Agronômico de Campinas, São Paulo, Brazil.

Cells of the *E. coli* strain Rosetta-gami 2 (DE_3_) pLysS (genotype: Δ(*ara–leu*)*7697* Δ*lacX74* Δ*phoA Pvu*II *phoR araD139 ahpC galE galK rpsL* (DE_3_) F’[*lac^+^lacI^q^ pro*] *gor522*::Tn*10 trxB* pLysSRARE_2_ (Cam^R^, Str^R^ and Tet^R^)) were acquired from Novagen, and cells of the *E. coli* strain JM109 (genotype: e14^−^(McrA^−^) *end*A1, *rec*A1, *gyr*A96, *thi-1, hsd*R17 (rk^–^, mk^+^), *rel*A1, *sup*E44, Δ(*lac-pro*AB) and [F′ *tra*D36 *pro*AB *laq*I^q^ZΔM15]) were acquired from Promega. Competent cells of these strains were chemically prepared as previously reported [14].

Animals: Isogenic female R/A Tor rats, generally high producers of IgE, Wistar rats and Balb/c mice were obtained from the animal facility of the Universidade Federal Fluminense, Niteroi, RJ, Brazil, and all experimental procedures were approved by the animal research ethics committee of this university (Proc. CEUA-UENF/297).

Serum containing IgE: Isogenic female R/A Tor rats were immunized with the 2S albumin pool, and the sera were separated as described by Felix et al. [13].

Anti-2S albumin serum: An anti-2S albumin from castor seeds was generated in rabbits as described [10].

### Native Ric c1 isolation

nRic c1 isolation: The 2S albumin pool (nRic c1 and nRic c3) was isolated and characterized by SDS/PAGE and immunoblotting as previously described [5], and the isoforms Ric c1 and Ric c3 were isolated as described by Felix et al. [13].

### Production of rRic c1

#### Insertion of the coding sequence of Ric c1 into the cloning vector

We designed a cloning strategy for amplifying the coding sequence from DNA based on an analysis of the 2S albumin gene from *R. communis*, without the removal of a linker peptide.

DNA was extracted from leaves of COP using the DNeasy Plant Mini Kit (Qiagen) following the manufacturer’s protocol. Ric c1 coding sequence was amplified by PCR from the extracted DNA using the forward primer 5′-CCAAGCCAGCAGGGGTG-3′ (*T*_m_: 64°C) and the reverse primer 5′-TTAGAACCGGCATTCGGTTG-3′ (*T*_m_: 63°C). The primers were designed based on the DNA sequence of the 2S albumin Ric c1 gene from *R. communis* (Gene ID: 8280732, National Center for Biotechnology Information (NCBI)). The annealing temperatures and capacities for self-complementarity of the primers were assessed using Primer-BLAST. PCRs were performed to amplify the coding sequence of Ric c1 (bp). Each reaction contained 10× *Pfu* buffer, 10 pmol/µl of each primer, 20 ng/μl DNA, 0.6 U *Pfu* DNA polymerase, 1.5 mM MgCl_2_ and 1.5 mM dNTPs. The cycling conditions were 95°C for 1 min followed by 40 cycles of 95°C for 45 s, 63°C for 45 s and 72°C for 3 min. The PCR product was cloned in *E. coli* using the pJET 1.2/blunt vector following the manufacturer’s protocol (CloneJET PCR cloning kit, Thermo Scientific). The PCR products were first blunt-ended following the manufacturer’s protocol using 75 ng of each PCR product and then incubated with 12.5 ng of the pJET 1.2/blunt vector and 0.5 U of T_4_ DNA ligase. The JM109 competent cells were transformed by heat shock with 4.5 µl of the vector containing the ligated PCR product, and the cells were maintained on ice for 10 min, transferred to 42°C for 90 s and then to ice for 10 min. Liquid LB medium (0.5% yeast extract, 1% tryptone and 1% NaCl) was added, and the cells were incubated at 37°C for 1 h. The cells were then plated on solid LB medium containing 100 μl/ml ampicillin and incubated at 37°C for 16 h for colony growth. Positive clones were analysed by plasmid extraction and digestion with the restriction enzyme *Bgl*II following the manufacturer’s protocol.

Primers for cloning Ric c1 into the pET-32 EK/LIC expression vector were designed following the manufacturer’s protocol (EK/LIC cloning Kit TB163, Novagen), which permitted directional cloning. The forward and reverse primers were 5′-**GACGACGACAAG***ATG*CCAAGCCAGCAGGGGTG-3′ and 3′-GTTGGCTTACGGCCAAGATT **TGGCCCGAAGAGGAG**-3′ respectively. The bold letters indicate the sequence that anneals to the coding sequence of Ric c1 DNA, and the underlined letters indicate the sequence that anneals to the vector, which were introduced to generate specific overhangs to anneal with the pET vector after treatment with T_4_ DNA polymerase. A Met codon, indicated by italics in the forward primer, was included as a requirement for cloning into the pET-32 EK/LIC vector. Each PCR contained 10× *Pfu* buffer, 10 pmol/µl of each primer, 10 ng/μl DNA, 0.6 U *Pfu* DNA polymerase, 1 mM MgCl_2_ and 1.25 mM dNTPs. The cycling conditions were 95°C for 1 min followed by 35 cycles of 95°C for 30 s, 74°C for 45 s and 72°C for 2 min, with a final extension at 72°C for 6 min. The amplified product was purified with a Wizard SV gel and a PCR clean-up system (A 9281, Promega) following the manufacturers’ protocols. The purified product was then treated with T_4_ DNA polymerase to create ends compatible with the vector following the manufacturer’s protocol.

The Ric c1 gene was inserted into the expression vector (pET-32 EK/LIC) following the manufacturer’s protocol (pET System Manual TB055, Novagen) and cloned into the JM109 competent cells. The positive clones were analysed by plasmid extraction and digestion with *Bgl*II and *Eco*RI following the manufacturer’s protocol.

The fragment-vector construct was named “Ric c1-pET” and was purified with the Wizard SV gel and the Promega PCR clean-up system. The purified construct was used to transform the Rosetta-gami 2 (DE_3_) pLysS competent cells by heat shock as described for the JM109 cells.

#### Production and purification of rRic c1

rRic c1 was produced following the method described by dos Santos et al. [15]. The Rosetta-gami 2 (DE_3_) pLysS host cells containing the Ric c1 clone were grown in LB medium containing 100 μg/ml ampicillin and incubated at 37°C with 250 rpm agitation to an OD_600_ of 0.5. After the growth stage, an aliquot of the culture was withdrawn for an uninduced control, isopropyl β-D-1-thiogalactopyranoside (IPTG) was added to the medium to a final concentration of 1 mM, and the cells were incubated at 37°C for an additional 3 h. The cells were harvested by centrifugation (15 000×***g*** for 10 min), re-suspended in phosphate buffer (50 mM sodium phosphate, pH 8.0, 300 mM NaCl) and lysed by sonication with five 30 s bursts in the presence of a cocktail of protease inhibitors (Sigma S8830). The cell lysate was clarified by centrifugation (15 000×***g*** for 10 min) and used for protein purification.

The recombinant proteins were initially purified by affinity chromatography on an Ni-NTA-agarose (nitrilotriacetic acid) column (Qiagen) as described by [10]. Protein was applied to the column previously equilibrated with phosphate buffer without the inhibitor cocktail. The resin was washed with five volumes of the same buffer containing 50 mM imidazole, followed by five volumes of the same buffer containing 500 mM imidazole. The retained sample was eluted with 500 mM imidazole, which contained Trx- and His-tagged rRic c1, and was treated with recombinant enterokinase (EK) (Sigma) following the manufacturer’s instructions. The proteins were analysed using 15% SDS/PAGE and immunoblotting [16]. For the blot assays, recombinant rRic c1 was spotted onto a nitrocellulose membrane and allowed to dry. The membrane was then incubated with total rabbit serum (1:50). Secondary anti-rabbit IgE (0.5 mg/ml) was diluted 1:2000 and then added to the membrane for 2 h. The colour of all probes was developed with a substrate mixture containing 5 mg of 3,3′-diaminobenzidine (DAB) in 4.9 ml of water, 300 ml of 0.1 M imidazole, 100 ml of 2 M Tris/HCl buffer (pH 7.5) and 5 ml of 30% H_2_O_2_.

rRic c1 was further purified using reversed-phase chromatography with a C18 column and solvents A (2% acetonitrile (ACN) and 0.1 trifluoroacetic acid (TFA)) and B (80% ACN and 0.1% TFA) in a gradient of 100% from 10 to 60 min at a flow rate of 0.7 ml/min. The peaks were collected after 10–20 min (not retained) and 33–35 min (retained), corresponding to Ric c1 with the Trx and His tags. The fractions were then treated with EK to remove the tags and contaminants. The peak corresponding to the retained proteins was submitted to the same chromatographic conditions described above, and rRic c1 (retention time of 26 min) was collected and sequenced by the Edman method [17].

### Production of mutant recombinant Ric c1 (mrRic c1) – engineering, cloning, expression and purification

**Figure 1:**
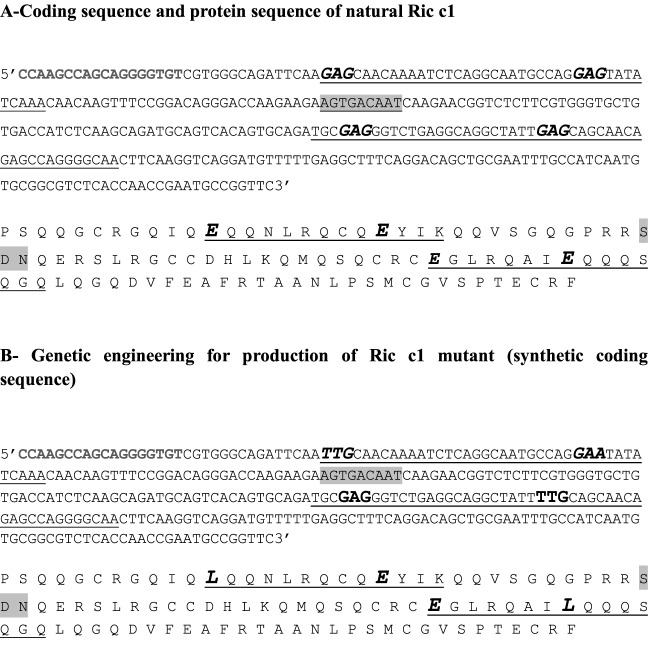
Ric c1 sequence codons (**A**) Ric c1 sequence, natural coding sequence without mutations. The region of the allergen epitopes is underlined. The Glu codons involved in allergic response are highlighted in bold and italics. The codons for the link peptide are highlighted in the grey box. The same highlights are shown for the amino acid sequence. (**B**) Mutated Ric c1 nucleotides and amino acids. The highlights are the same as in (A).

The mrRic c1 coding sequence was designed using GeneArt (Invitrogen). The sequence of the Ric c1 gene from *R. communis* was provided by Gene ID 8280732 from NCBI, and specific codons for glutamic acid residues were mutated to codons for leucine residues. Only one of the two glutamic acid residues in each allergen epitope was mutated (Figure 1A). The glutamic acid residues involved in α-amylase inhibition, as predicted by *in silico* studies by Nascimento et al. [8], were conserved. GeneArt was used for the synthesis. The synthetic coding sequence was produced, sequenced, and cloned into the cloning vector pMA-T (Figure 1B and Supplementary Figures S1 and S2) to produce the construct pMA-T-mrRic c1. pMA-T-mrRic c1 was transformed into *E. coli* strain XL-10 by heat shock to propagate and conserve this vector in a biological system. The transformed clones were analysed by plating the cells on LB solid medium containing 100 µg/ml ampicillin and 34 µg/ml chloramphenicol. Colonies were collected and grown in LB medium with the same antibiotics, and the transformed XL-10 cells were stored at −70 °C until needed.

pMA-T-mrRic c1 was used as a template for PCR to amplify mrRic c1 containing the regions that bind to the pET-32 EK/LIC expression vector, producing the construct mrRic c1-pET. The PCR conditions, the primer sequences and the construct Ric c1-pET cloned into the Rosetta-gami 2 (DE_3_) pLysS expression host were the same as those described in section “Insertion of the coding sequence of Ric c1 into the cloning vector”.

Each of three positive Rosetta-gami_2_ (DE_3_) pLysS colonies was transferred to 5 ml of LB medium containing 100 µg/ml ampicillin and 34 µg/ml chloramphenicol for the induction of mrRic c1. The cultures were incubated at 37°C under agitation at 250 rpm to an OD_600_ of approximately 0.5, and 300 µl were transferred to 10 ml of fresh LB medium containing the same antibiotics. These cultures were incubated under the same conditions described above to an OD_600_ of approximately 0.5 and aliquoted equally (5 ml each). One aliquot received 1 mM IPTG to induce mrRic c1 expression, and the other aliquot served as an uninduced control. mrRic c1 was produced and purified as described in Section “Production and purification of rRic c1” for rRic c1.

### Characterization of recombinant proteins – SDS/PAGE and immunodetection

The cells were harvested by centrifugation (15 000×***g*** for 10 min), re-suspended in phosphate buffer (50 mM sodium phosphate, pH 8.0; 300 mM NaCl) and lysed by adding CelLytic B, following the instruction manual (Sigma-Aldrich), in the presence of a cocktail of protease inhibitors (Sigma). The cell lysate was clarified by centrifugation (15 000×***g*** for 10 min). The expression of rRic c1 and mrRic c1 was initially analysed by 15% SDS/PAGE [18]. The unpurified bacterial lysate (15 µl), including both the expressed protein and all *E. coli* proteins, and 15 µl of the bacterial culture were submitted to 15% SDS/PAGE. The presence of rRic c1 or mrRic c1 in the induced fraction was confirm by Western blotting [16]. The SDS/PAGE gel was transferred to a nitrocellulose membrane and blotted in a semi-dry system of filter papers soaked in transfer buffer (20 mM Tris, glycine 145 mM, 20% methanol) under a current of 1 mA for each cm^2^ of membrane for 2 h. The membrane was then soaked in blocking buffer (0.1 M Na_2_HPO_4_, 0.15 M NaCl, 2% skim milk powder) for 1 h. The blocking buffer was exchanged for the first antibody, an anti-2S albumin from castor seeds generated in rabbits [10]. The membrane was incubated with the primary polyclonal antibody at 4°C for 18 h. The membrane was washed ten times for 5 min each in PBS (0.1 M Na_2_HPO_4_, 0.15 M NaCl). The membrane was then incubated with the secondary antibody, an anti-rabbit IgG conjugated to peroxidase (Sigma-Aldrich) diluted with blocking buffer. The colour of all probes was developed with a substrate mixture of 5 mg of DAB in 4.9 ml of water, 300 ml of 0.1 M imidazole, 100 ml of 2 M Tris/HCl buffer (pH 7.5) and 5 ml of 30% H_2_O_2_.

### Biological tests

#### α-Amylase inhibitory assays

α-Amylase inhibitory assays (EC 3.2.1.1) using *C. maculatus* α-amylase were performed using the method described by Bernfeld (1955) and modified by Nascimento et al. [8]. Enzymes (25 μg/ml in 0.01 M phosphate buffer, pH 5.5) were assayed using a 1% starch substrate. The reaction was stopped by adding 3,5 dinitrosalicylic acid at 100°C and monitored at 540 nm. Each assay contained 10 U of α-amylase. The α-amylase was incubated with 10 µg of nRic c1, rRic c1 or mrRic c1 in a water bath at 37°C for exactly 15 min before the substrate solution was added (1% starch). The assay proceeded for 30 min. All inhibition assays were performed in triplicate.

#### Allergy evaluation

We evaluated the ability of mrRic c1 to modulate the onset of allergic response using a mast-cell degranulation assay and analysing antibody profiles for total IgG, IgG1 and IgE produced after immunization.

##### Mast-cell degranulation assay

Rat mast cells were isolated as described by Deus-de-Oliveira et al. [10]. Wistar rats (250 g) were killed with CO_2_, and a peritoneal wash was performed by injecting 20 ml of Dulbecco’s Modified Eagle Medium (DMEM) containing 12 U/ml heparin. The abdomen was gently massaged for approximately 90 s. The peritoneal cavity was carefully opened, and the fluid containing peritoneal cells was aspirated with a Pasteur pipette. The cells were transferred to Petri dishes and incubated at 37°C for 30 min. Two-thirds of the supernatant was aspirated and discarded. The mast cells were separated into aliquots of 100 µl (approximately 1.0 × 10^4^ cells) and kept at room temperature. Rat peritoneal mast cells (100 µl) were incubated with rat pre-immune serum as a negative control and with the serum of a rat previously immunized with 2S albumin. The sera of R/A Tor rats, high producers of IgE, were used in these experiments as a source of IgE (2S alb rat-IgE). The cells were sensitized with anti-2S alb rat-IgE, washed twice with DMEM and then incubated with the sample. Each experiment was performed in the presence or absence of 100 ng of Ric c1, rRic c1 or mrRic c1. The extent of mast-cell degranulation was determined after incubation with 2S alb rat-IgE (R/A Tor rat serum) and subsequent exposure to nRic c1, rRic c1 or mrRic c1 [10]. Briefly, the cells (10 µL) were stained for 15 min with 10 µl of 0.1% Toluidine Blue containing 10% formaldehyde and 1% acetic acid, pH 2.8, to visualize the degranulated mast cells. The granulated and degranulated mast cells were counted under a light microscope at 40× in a Neubauer chamber.

##### Analysis of antibody profiles during immunization

Balb/c mice were divided into three groups (two mice per group): the first group (G1) was sensitized with phosphate buffer saline (PBS) and named G1-PBS, the second group was sensitized with native Ric c1 (G2-nRic c1) and the third group was sensitized with mutant recombinant Ric c1 (G3-mrRic c1). Briefly, mice were sensitized with 0.1 μg of nRic c1 (G2) or mrRic c1 (G3) + 2 mg aluminium hydroxide in 100 μl of PBS on day 0, 15 and 30. The G1-PBS group was sensitized with 100 μl of PBS + 2 mg aluminium hydroxide on the same days. Animals were bled seven days after each immunization. Total IgG, IgG1 and IgE were determined using an enzyme-linked immunosorbent assay (ELISA). A 96-well plate (Nunc Immuno) was sensitized with 0.33 μg of 2S albumin of castor oil seeds, incubated for 18 h, and then washed twice with PBS, pH 7.0. A blocking solution containing 1% gelatin in PBS and Tween 20 in 200 μl was incubated for 1 h, washed with PBS and then incubated with the primary antibody at a ratio of 1:500 for 1 h at 37°C, followed by further two washes. Finally, it was incubated with the secondary antibody (IgE at 1:200, IgG at 1:500 and IgG1 at 1:100) for 1 h at 37°C. A peroxidase kit was used for detection.

### Protein sequencing

#### N-terminal sequence

The N-termini were sequenced by automatic Edman degradation using a PPSQ-33A Protein Sequencer (Shimadzu) [17].

#### Mass spectrometry

Mass spectrometric analysis of mrRic c1 was carried out in a nanoACQUITY UPLC columns connected to a Synapt G2-Si HDMS mass spectrometer (Waters, Manchester, U.K.). Samples were loaded onto a nanoACQUITY UPLC 5 µm C18 trap column (180 µm × 20 mm) at 5 µl/min for 3 min and then onto a nanoACQUITY HSS T3 1.8 µm analytical reversed-phase column (100 µm × 100 mm) at a flow rate of 500 nl/min. Peptides were eluted using a binary gradient, with mobile phase A consisting of water (Tedia, Fairfield, U.S.A.) and 0.1% formic acid (Sigma-Aldrich) and mobile phase B consisting of ACN (Sigma-Aldrich) and 0.1% formic acid (Sigma-Aldrich). The gradient sequence was 7–40% B from 0 to 33.21 min, 40–85% B from 33.21 to 37.21 min, 85%-85% B from 37.21 to 41.21 min and 85–7% B from 41.21 to 43.21 min. Mass spectrometry was performed in positive resolution mode (V mode) and in data-independent acquisition mode. Transfer collision energy ramped from 20 to 35 V in high-energy mode, cone and capillary voltages were 30 and 2800 V respectively, and the source temperature was 60°C. Spectral-acquisition scan rates were set to 0.5. Human [Glu1]-fibrinopeptide B (Sigma-Aldrich) at 100 fmol/μl was used as an external calibrating standard. Spectral processing and database searches used Protein Lynx Global Service v.3.02 (Waters) with the following parameters: Min Fragment Ion Matches per Peptide, 2; Min Fragment Ion Matches per Protein, 5; Missed Cleavages, 2; Fixed Modifier Reagents per carbamidomethyl, C; and Variable Modifier Reagent Oxidation, M.

## Results

### Production of rRic c1 in *E. coli*

Ric c1 and Ric c3, two 2S albumin isoforms, are important allergens in the seeds and pollen of castor oil plants. Ric c3 and Ric c1 are produced from the N- and C-termini respectively, of the same polypeptide precursor. They are both post-translationally modified. A linked serine–aspartic–asparagine (SDN) peptide is released from the pre-processed precursor during Ric c1 production, forming native Ric c1 (nRic c1), which contains two polypeptide chains linked by disulphide bonds. Recombinant Ric c1 (rRic c1) was produced as a single chain containing the SDN link peptide.

Fifteen transformed colonies with the pJET 1.2/blunt vector ligated to the coding sequence of Ric c1 were grown in LB culture medium containing ampicillin and the plasmids were extracted, digested, and visualized by electrophoresis in a 1% agarose gel. The size of the fragment released from the digested plasmid was compared with the size of the PCR product coding for Ric c1 (300 bp, arrow in Supplementary Figure S1A). A PCR temperature gradient was performed, and 74°C was chosen (Supplementary Figure S1B). *E. coli* Rosetta-gami 2 (DE3) pLysS cells harbouring the pET-32 EK/LIC plasmid and control cells were cultured as described above.

**Figure 2:**
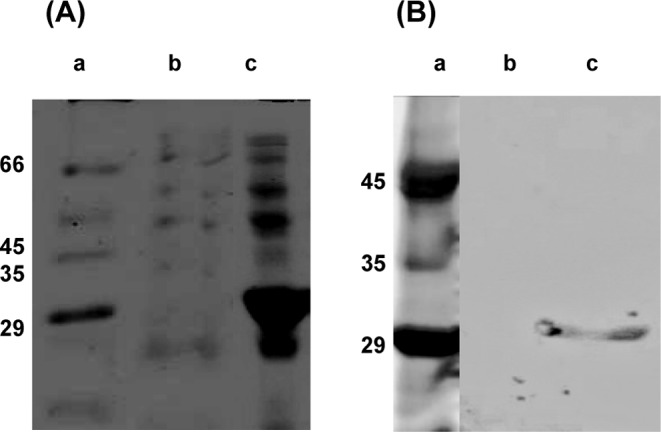
Recombinant Ric c1 production 15% SDS/PAGE and Western blot of the rRic c1 construct (**A**) 15% SDS/PAGE: Lane a, protein marker; lane b, crude extract of *E. coli* Rosetta-gami 2 (DE3) pLysS lysate control (without induction); lane c, protein extracted from induced *E. coli* Rosetta-gami 2 (DE_3_) pLysS cells containing the construct Ric c1-pET. (**B**) Western blot using polyclonal antibodies raised against the 2S albumin pool. Lane a, molecular-weight marker; lane b, *E. coli* Rosetta-gami 2 (DE_3_) pLysS/Ric c1-pET lysate cells (without induction); lane c, rRic c1 purified by affinity (Ni-NTA) chromatography.

**Figure 3:**
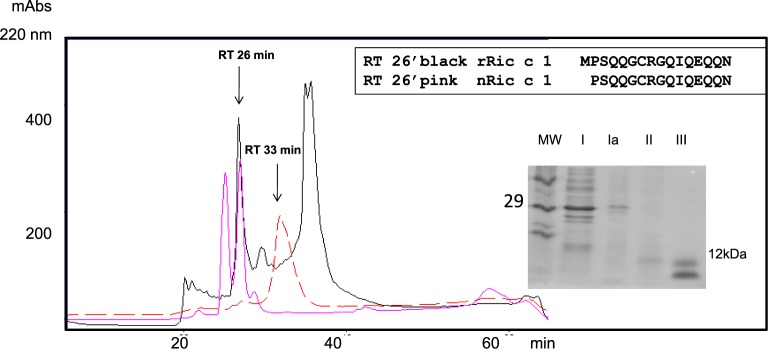
Recombinant Ric c1 isolation – reversed-phase high-performance liquid chromatography (RP-HPLC) on a C18 column Elution: solvent A (2% ACN and 0.1% TFA) and solvent B (80% ACN and 0.1% TFA) in a gradient of 100% from 10 to 60 min at a flow rate of 0.7 ml/min. The pink line represents the RP-HPLC profile of the 2S albumin pool (RIC c1 + Ric c3). The retention time (RT) of nRic c1 was 26 min. The Trx-tagged rRic c1 isolated by affinity chromatography (Ni-NTA) was purified by RP-HPLC (red line). The protein in the broad peak (31–34 min) was isolated, cleaved with EK and purified under the same conditions. The peak for the EK-treated sample with a retention time at 33 min disappeared, and a peak with retention time at 26 min appeared (black line). Inset: partial N-terminal sequence of the protein isolated at 26 min, and the SDS/PAGE of the fractions obtained during purification. I, induced *E. coli* extract; Ia, protein isolated after affinity- and RP-HPLC; II, protein isolated at 26 min; III, 2S albumin pool (Ric c1 + Ric c3). Note the Met codon added in the sequence of rRic c1 as a cloning requirement.

The Ric c1 coding sequence inserted into the pET-32 EK/LIC vector was produced in *E. coli* fused with the linker peptide containing Trx and His tags. SDS/PAGE for both proteins, from the control cells and the Rosetta-gami 2 (DE_3_) pLysS/Ric c1-pET transformants (Figure 2A, lanes b and c), indicated that the transformed cells produced a 29-kDa major protein (Figure 2A, lane c). This distinct band was not observed at the corresponding position in the control (Figure 2A, lane b). The production of Ric c1 as the induced 29-kDa protein, after purification by affinity chromatography on a Ni-NTA column, was confirmed by immunoblotting (Figure 2B, lane c). The purification of rRic c1 was improved by adding liquid chromatography on a reversed-phase HPLC system. His-Trx-tagged rRic c1 was eluted at 33 min (Figure 3, red peak). This protein was cleaved by EK and purified by reversed-phase C18 HPLC. A peak eluted at 26 min, probably rRic c1 (Figure 3, black line) overlapping with nRic c1 (Figure 3, pink line), was isolated and characterized. The collected fractions were visualized by SDS/PAGE (inset Figure 3). The N-terminal sequence (inset Figure 3) of the fraction collected at 26 min (pink) confirmed that the coding sequence of Ric c1 had been expressed and that rRic c1 was produced (black). Note the Met codon added in the sequence of rRic c1 as a cloning requirement.

### Production of mrRic c1 in *E. coli*

**Figure 4:**
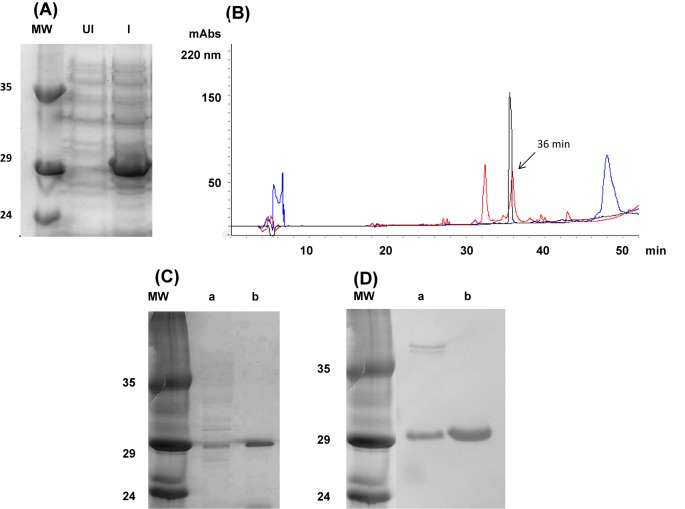
Mutant recombinant Ric c1 isolation and characterization **(A)** 15% SDS-PAGE profile: MW, Molecular Weight Marker; UI, uninduced crude *E. coli* extract and I, induced crude *E. coli* extract. **(B)** Reversed-phase chromatography on a C18 column (RP-HPLC): Elution: solvent A (0.1% TFA) and solvent B (80% ACN and 0.1% TFA) in a gradient of 100% from 10 to 60 min at a flow rate of 0.7 ml/min. The red line represents the RP-HPLC profile of the 2S albumin pool (Ric c1 + Ric c3). The retention time (RT) of nRic c1 was 36 min. The blue line represents the chromatogram of crude induced mutant-recombinant extract purified directly by RP-HPLC. The protein in the broad peak (47–50 min) was isolated, purified under the same conditions and cleaved with EK. The EK hydrolysed product was purified by RP-HPLC under the same conditions and mrRic c 1 was eluted at 36 min (black line). **(C)** 15% SDS/PAGE : MW, protein marker; lane a, crude induced *E. coli* extract; lane b,Trx-tagget mrRic c1 (C18 HPLC broad peak 47-50 minutes. **(D)** Western blot using primary antibodies raised against the 2S albumin in rabbit. MW, molecular-weight marker; lane a, crude induced *E. coli* extract; lane b, Trx-tagget mrRic c1 (C18 HPLC broad peak 47-50 minutes.

The coding sequence, determined by Invitrogen, confirmed the exchange of glutamic acid residues with leucine residues at positions 66 and 200 of mrRic c1 (highlighted in the box, Suplementary Figures S2 and S3). The expression of mrRic c1 by the *E. coli* Rosetta-gami 2 (DE_3_) pLysS cells transformed with the expression vector pET-32 EK/LIC ligated to the mrRic c1 coding sequence was demonstrated. SDS/PAGE indicated that the induced (I) *E. coli* Rosetta-gami 2 (DE_3_) pLysS cells produced a 29-kDa protein band (Figure 4A, lane I) and that the uninduced cells (UI) did not (Figure 4A, lane UI). The chromatographic profile of the purification of mrRic c1 is presented in Figure 4B overlapped with the 2S albumin pool (nRic c1 and nRic c3) under the same elution conditions. The chromatographic profile of nRic c1 (Figure 4B, red line), used as a control, contained a peak with a retention time of 36 min. The chromatographic profile of the crude induced extract indicated a broad peak eluted between 47 and 50 min (Figure 4B, blue line). This peak was collected, concentrated and treated with EK. The profile obtained after enzymatic hydrolysate fractionation is shown by the black line in Figure 4B. A peak eluted at 36 min, near nRic c1, which was probably mrRic c1, was collected and further characterized. The SDS/PAGE profile is shown in Figure 4C (line a is the crude induced extract, and line b is the purified broad peak at 47–50 min). Figure 4D shows the immunodetection of these peaks, using the anti-albumin 2S primary antibody generated in rabbits. Western blotting provided evidence that the purified protein was mrRic c1. The purified peak, isolated by reversed-phase chromatography (black peak, with retention time of 36 min) was characterized by mass spectrometry.

Table 1 presents the molecular weights and sequences of the tryptic peptides identified by mass spectrometry and the overlap of these peptides with the predicted mrRic c1 sequence. The coverage of the primary structure of mrRic c1 was nearly complete; only the N-terminal peptide (MPSQQGCR) was not identified by mass spectrometry (Table 1) but was identified by Edman degradation. We compared this result with the recombinant protein predicted from Sulp. 1 to certify the exchange of glutamic acid residues with leucine residues and that unwanted mutations were not incorporated in mrRic c1.

**Table 1:** Tryptic peptides identified by mass spectrometry

Peptide number	Molecular weight	Peptide sequence
**P1**	2147.1	GQIQLQQNLRQCQ**E**YIK
**P2**	1889.9	QCQEYIKQQVSGQGPR
**P3**	1112.6	QQVSGQGPRR
**P4**	748.3	SDNQER
**P5**	1245.6	SLRGCCDHLK
**P6**	1807.7	GCCDHLKQMQSQCR
**P7**	3134.5	C**E**GLRQAILQQQSQGQLQGQDVFEAFR
**P8**	1997.9	TAANLPSMCGVSPTECRF
Overlapping of tryptic peptides, identified by mass spectrometry, on mrRic c1 sequence.		
Complete primary strucutre of mrRic c1: *,†		
***PSQQGCR***/GQIQLQQNLRQCQ**E**YIK/QQVSGQGPRR/*SDN*QER/SLRGCCDHLK/QMQSQCR/C**E**GLRQAILQQQSQGQLQGQDVFEAFR/TAANLPSMCGVSPTECRF		

Highlighted in **bold** are glutamate residues (**E**) involved in IgE binding, and highlighted as underlined are leucine residues (L).

*The N-terminal ***PSQQGCR*** (***bold-italic***) peptide was not identified by mass spectrometry.

†The linked peptide *SDN* is highlighted in *italic*.

### Biological activity

#### Mast-cell degranulation and antibodies profile

**Figure 5:**
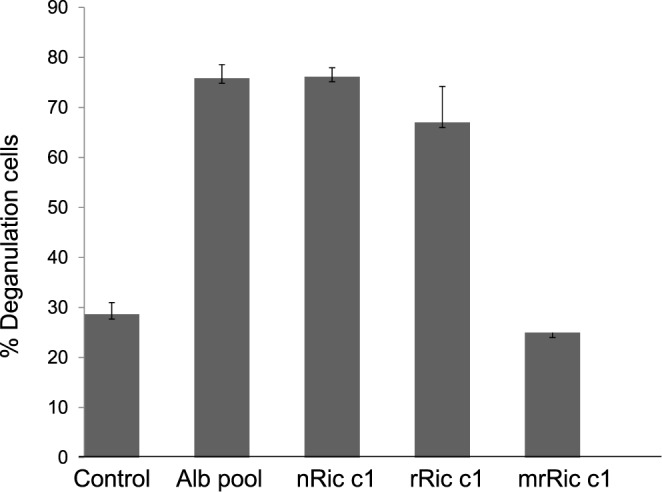
Allergenic activity – mast-cell degranulation assay Control mast cells were incubated only with the anti-2S albumin serum. Alb-pool mast cells were previously incubated with the anti-2S albumin serum exposed to a 2S albumin pool. nRic c1, rRic c1, and mrRic c1: mast cells sensitized with the anti-2S albumin serum and exposed to nRic c1, rRic c1 and mrRic c1 respectively.

**Figure 6:**
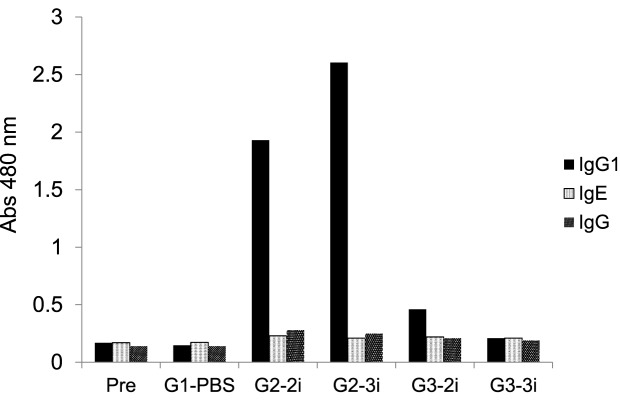
Serum immunoglobulin profile Pre, pre-immune serum; G1-PBS, negative control (mice with intraperitoneal injection of phosphate buffer); G2-2i and G2-3i, mice immunized with rRic c1; G3-2i and G3-3i, mice immunized with mrRic c1. The means for two mice per group are shown.

Figure 5 shows that the 2S albumin pool, nRic c1, and rRic c1 induced 76, 75.8, and 67% mast-cell degranulation, demonstrating that the allergenic characteristics of rRic c1 were similar to those of the native protein. These results indicated that the biological activity of rRic c1 synthesized in prokaryotic cells as a single chain was similar to that of nRic c1 isolated from castor oil seeds. mrRic c1 induced only 25% mast-cell degranulation, similar to the mast-cell negative control. The antibody profile also indicated that the levels of IgG1 produced in animals sensitized with rRic c1 (G2) were high, and the levels of IgG1 produced after mrRic c1 sensitization (G3) were similar to those obtained for the control animals (G1) (Figure 6). The mast-cell degranulation assay and the IgG profile supported the *in silico* studies by Nascimento et al. [8] and demonstrated that the mutant was a hypoallergenic protein.

#### α-Amylase inhibition

**Figure 7:**
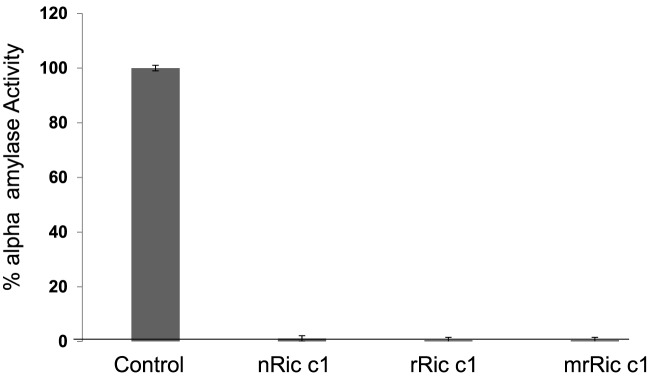
α-Amylase inhibition activity: effect of nRic c1, rRic c1, and mrRic c1 on *C. maculatus* α-amylase Enzymes and inhibitors were pre-incubated for 15 min prior to the addition of the substrate (1% starch) for measuring enzymatic activity. Inhibition (%) was relative to the control assay (without Ric c1). Each point is the average of three measurements.

nRic c1, rRic c1, and mrRic c1 inhibited 100% of the activity of *C. maculatus* α-amylase (Figure 7). These results indicated that mrRic c1 and rRic c1 synthesized in prokaryotic cells had biological activities similar to nRic c1 isolated from COP. These results therefore indicated that mrRic c1 retained a defensive role.

## Discussion

Ric c1 and Ric c3, two 2S albumin isoforms, are from same polypeptide precursor. They are both post-translationally modified, contain two polypeptide chains linked by disulphide bounds, and are occasionally glycosylated. Their allergenic properties pose health risks to farm workers and distributors of castor seeds. Castor oil is often used in a variety of industries. An increase in planting, however, has become a concern, despite the advantages of castor oil. A protein by-product after oil extraction known as castor cake contains ricin, a toxin and a set of allergenic proteins resistant to thermal detoxification. The allergens have also been described from the pollen [3]. Increased awareness of the dangers of contact with or inhalation of the castor allergens is thus necessary due to the increase in cultivation for commercial use.

Castor 2S albumin can cross-react with allergens from shrimp, fish, corn, wheat, soybean, peanut, house dust, tobacco and airborne fungi [10]. Exposure to the pollen or seeds can sensitize an individual and can trigger an allergic response when exposed to other foods. Obtaining standardized crude extracts of allergens for therapeutic purposes is difficult and may expose individuals to other substances in the seeds, thus contributing to non-specific allergenic responses. A linked “SDN” peptide is released from the pre-processed precursor during Ric c1 production, forming nRic c1. We produced a recombinant Ric c1, rRic c1, in *E. coli* using two purification steps. The 6× His-tagged recombinant protein was first purified by a Qiagen kit, a methodology revised and verified by Karakus et al. [19]. The His tag was enzymatically cleaved, and the protein was further purified by reversed-phase chromatography. rRic c1, produced as a single chain, preserved the allergenic properties and α-amylase inhibition of the native allergen. The cloning and highly effective expression of recombinant genes encoding the allergens without post-transcriptional modifications could be an important strategy for identifying allergenic properties, not only for castor allergens, but also for other allergens formed by two chains from a precursor polypeptide [20]. This strategy may contribute to the selection and acquisition of highly purified allergens for the development of an ASIT.

We also described the production of a hypoallergenic recombinant Ric c1 based on *in silico* molecular models suggesting that mutating specific glutamic acid residues to leucine residues would decrease the allergenicity of Ric c1 and Ric c3 but retain their inhibition of a-amylase activity [9]. This *in silico* study found that mutations in these allergenic epitopes altered the interaction with IgE and thus might not lead to the onset of allergic symptoms. The identification of allergenic epitopes has been used as a basis for the production of hypoallergenic proteins. Valenta et al. [21,22] described the importance of altering the structures of allergens for developing new forms of allergen-specific immunotherapy. Chen et al. [23] demonstrated that recombinant hypoallergenic hybrid proteins could be produced using synthetic genes coding for two hybrid proteins, consisting of reassembled Der p1 and Der p2 fragments with (recombinant Der p2 [rDer p2]/1C) and without (rDer p2/1S) cysteines, expressed in *E. coli*, and purified to homogeneity by affinity chromatography. Bogh et al. [24] demonstrated that mutations at aspartic acid residues in *Brassica rapa* allergens decreased IgE cross-linking in the membranes of sensitized mast cells, which decreased the allergenic potential of the protein [24]. Bouaziz et al. [25] produced a hypoallergenic mutant Cyp (mcyp) for immunotherapy for a food-specific allergy. This theoretical framework supported by the production of genetically modified recombinant proteins has emerged as a new approach for immunotherapies to prevent allergic reactions. We have produced recombinant 2S albumin isoforms and mutants in a bacterial expression system by exchanging glutamic acid residues with leucine residues.

In conclusion, we produced a recombinant Ric c1 as a single chain that retained both allergenicity and α-amylase inhibition and a mutant recombinant Ric c1 that retained the defensive function but was not recognized by specific IgE. Mutant constructs with linker peptides can be important for studying the allergenic properties of not only castor oil plant allergens, but also allergens from other sources. The point mutations we used support our continuing efforts to produce transgenic hypoallergenic COP and to develop an immunotherapeutic agent for allergy prevention.

## Supporting information

**Supplementary Figure 1:** Plasmid characterisation: (A) Electrophoretic profile of the plasmids extracted from JM109 cells transformed with the vector pJET containing the Ric c1 coding sequence and digested with *BglII*. Lanes 1 and 2, undigested plasmid; lane M, standard molecular-weight marker; lanes 2-16, digested plasmids; lane 17, the 300-bp Ric c1 PCR product used as control (arrow). A fragment of approximately 300 bp was released in several samples. (B) Gradient PCR for the temperature required to connect the Ric c1 coding sequence to the vector pET-32 EK/LIC. A temperature of 74 °C was chosen as the best condition.

**supplementary Figure 2:** Vector map Map of the pMA-T vector provided by Invitrogen containing the insertion region of the synthetic coding sequence for Ric c 1 (Ric _c_1_new), ampicillin resistance regions (AmpR), and the origin of replication (Col E1 origin).

**supplementary Figure 3:** Nucleotide sequence Nucleotide sequence of mrRi c1 cloned in pMA determined by Invitrogen (GeneArt). The arrows indicate the start and end of the mrRic c1 coding sequence.

## Abbreviations

ASIT: allergen*-*specific immunotherapy
COP: castor oil plant
COS: castor oil seeds
DAB: 3,3′-diaminobenzidine
EK: enterokinase
IPTG: isopropyl β-D-1-thiogalactopyranoside
mrRic c1: mutant recombinant Ric c1
nRic c1: native Ric c1
rRic c1: recombinant Ric c1
