# Supplementary material for: A modified, hypoallergenic variant of the Ricinus communis Ric c1 protein retains biological activity
Source: Biosci Rep. 2018 Mar 16;38(2):BSR20171245. doi: 10.1042/BSR20171245 (PMC5857906; doi:10.1042/BSR20171245)
Supplement: Supplementary file 1 [file bsr20171245_Supp1.pdf]

Supplementary Figures
Supplementary Figure 1

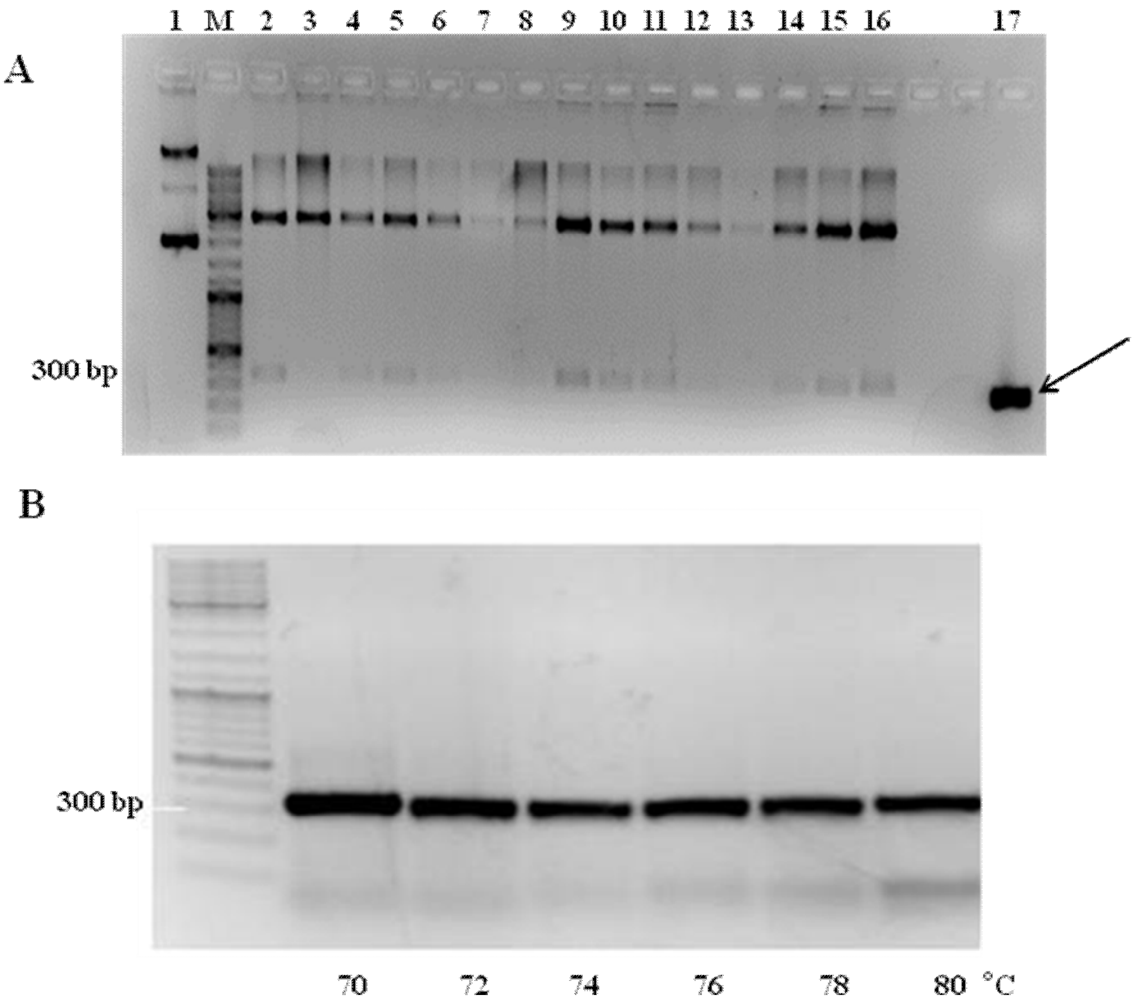

-

Plasmid characterisation:

(A) Electrophoretic profile of the plasmids extracted from JM109 cells transformed with the vector pJET containing the Ric c1 coding sequence and digested with *Bgl*II. Lanes 1 and 2, undigested plasmid; lane M, standard molecular-weight marker; lanes 2-16, digested plasmids; lane 17, the 300-bp Ric c1 PCR product used as control (arrow). A fragment of approximately 300 bp was released in several samples. (B) Gradient PCR for the temperature required to connect the Ric c1 coding sequence to the vector pET-32 EK/LIC. A temperature of 74 °C was chosen as the best condition.

Supplementary Figure 2

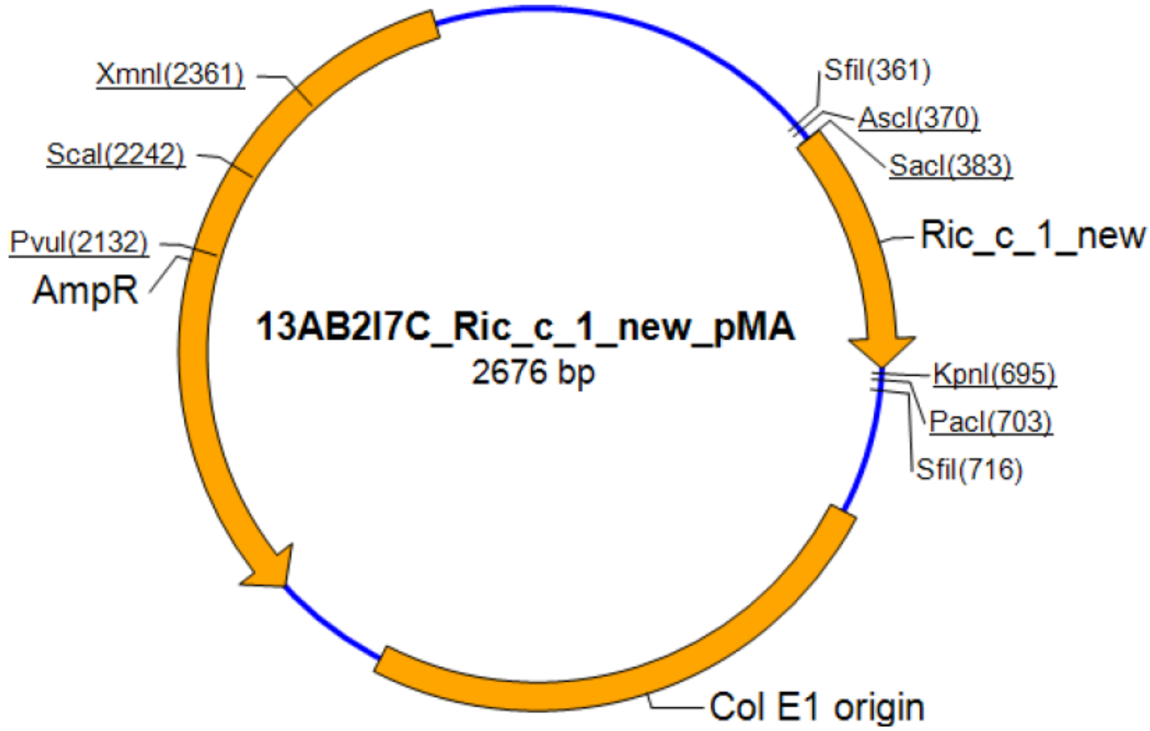

Vector map

Map of the pMA-T vector provided by Invitrogen containing the insertion region of the synthetic coding sequence for Ric c 1 (Ric _c_1_new), ampicillin resistance regions (AmpR), and the origin of replication (Col E1 origin).

Supplementary Figure 3

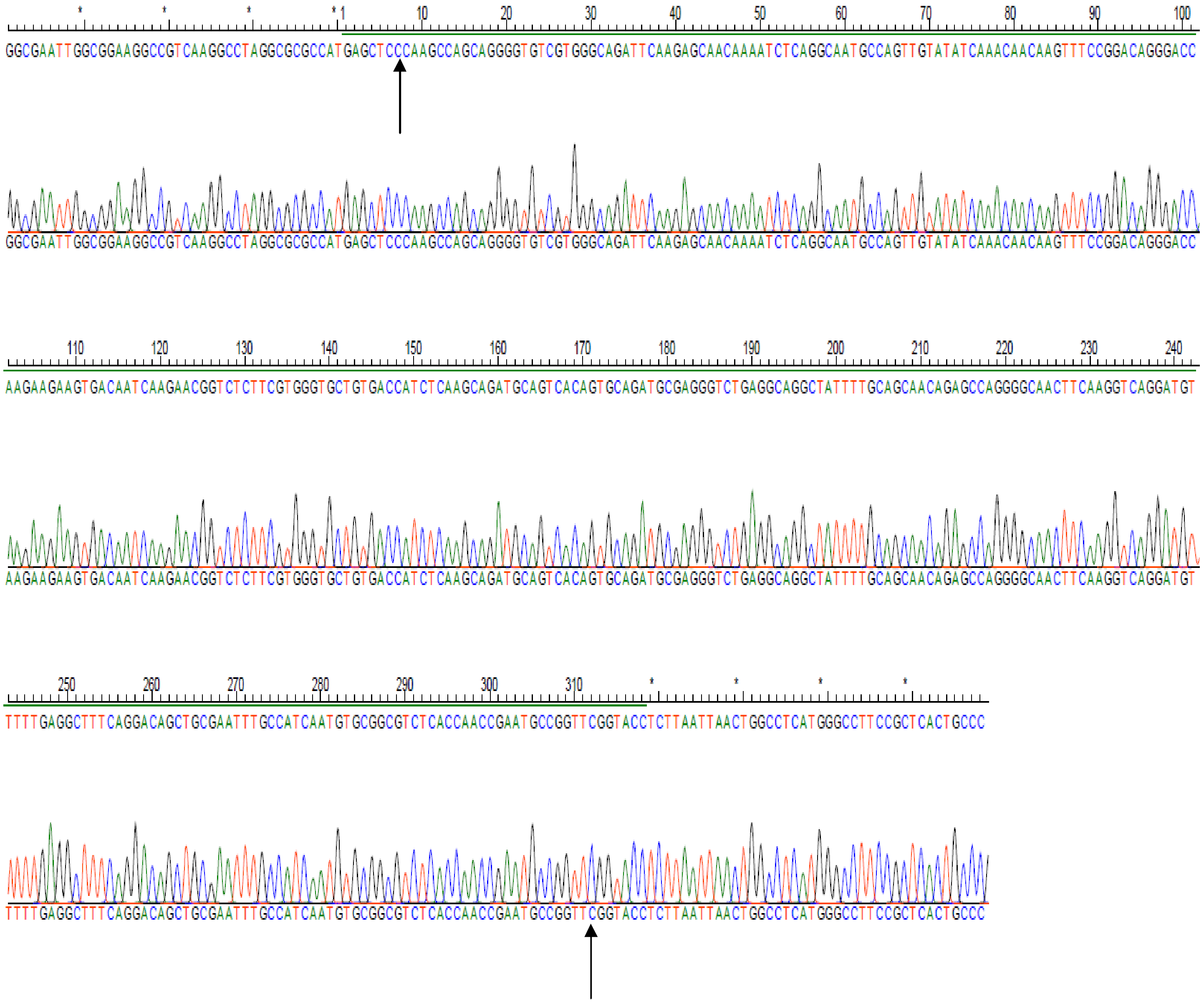

Nucleotide sequence

Nucleotide sequence of mrRi c1 cloned in pMA determined by Invitrogen (GeneArt). The arrows indicate the start and end of the mrRic c1 coding sequence.
